# Supplementary figures and images for: The epidemiology and disease burden of congenital TORCH infections among hospitalized children in China: A national cross-sectional study
Source: PLoS Negl Trop Dis. 2022 Oct 14;16(10):e0010861. doi: 10.1371/journal.pntd.0010861 (PMC9604879; doi:10.1371/journal.pntd.0010861)

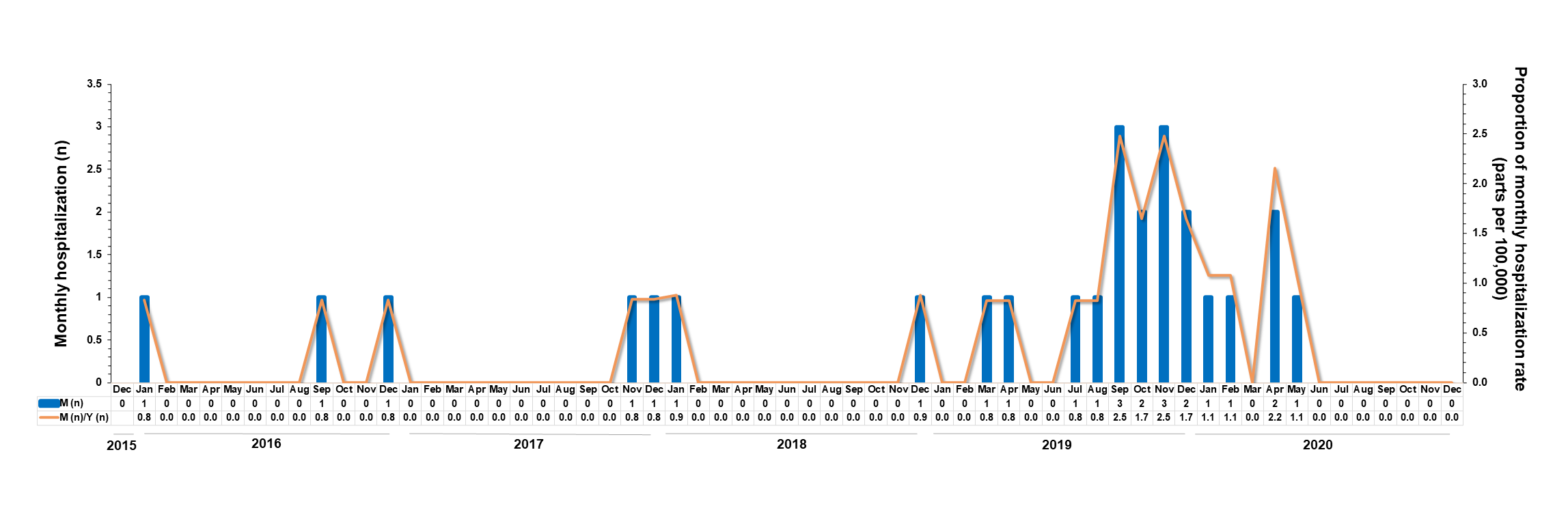

Supplement: S1 Fig — M (n): number of monthly hospitalizations, Y (n): number of yearly hospitalizations. (TIF) [file pntd.0010861.s001.tif]

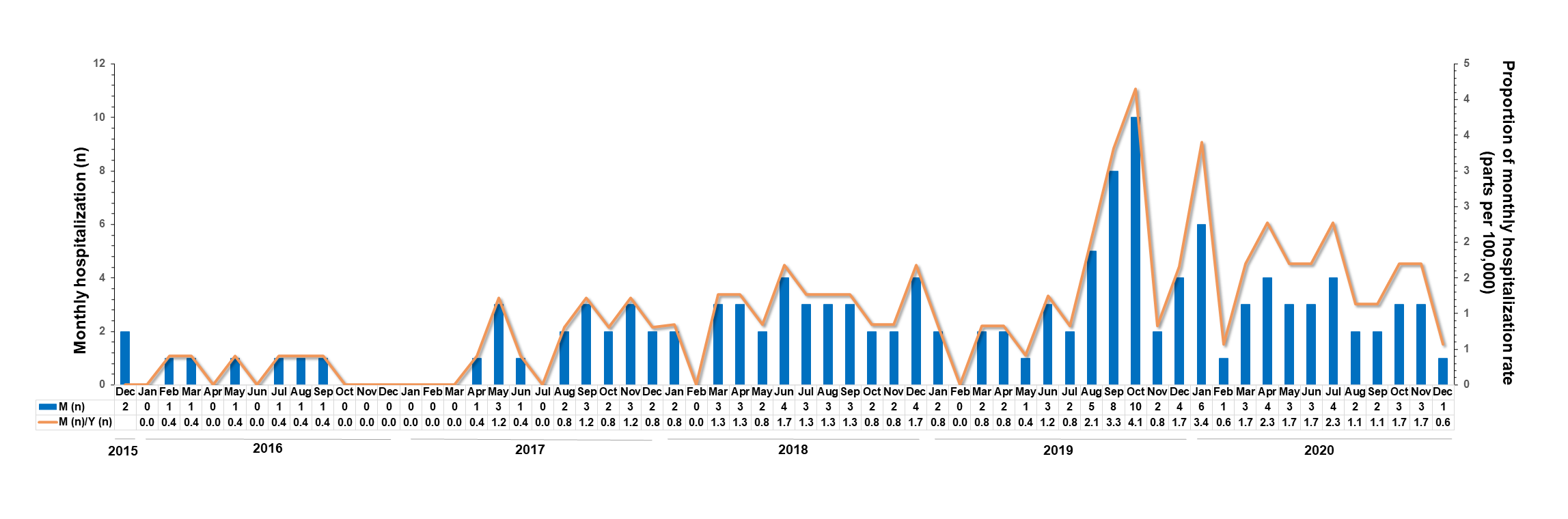

Supplement: S2 Fig — M (n): number of monthly hospitalizations, Y (n): number of yearly hospitalizations. (TIF) [file pntd.0010861.s002.tif]
