# Supplementary material for: The epidemiology and disease burden of congenital TORCH infections among hospitalized children in China: A national cross-sectional study
Source: PLoS Negl Trop Dis. 2022 Oct 14;16(10):e0010861. doi: 10.1371/journal.pntd.0010861 (PMC9604879; doi:10.1371/journal.pntd.0010861)
Supplement: Attachment — Submitted filename: Authors Response to Reviewers Comments.docx [file pntd.0010861.s003.docx]

**Point-by-point Response Letter**

***PLOS Neglected Tropical Diseases***

_____________________________________________________________________

Dear Editors Dr. Winsley Rose and Dr. Marcelo Ferreira,

Thank you for your decision letter concerning our manuscript (ID PNTD-D-22-00698)

entitled "The epidemiology and disease burden of congenital TORCH infections among hospitalized children in China: A national cross-sectional study", and the time allowed for our revision. I also appreciate all the critical comments from you and the reviewers. We have carefully considered the comments and revised the manuscript accordingly. With these improvements, we hope that the current version now meets the journal’s standards for publication. Point-by-point responses to all those comments and a list of changes we have made to the manuscript are provided below.

Sincerely yours,

Corresponding author:

Ran Wang (randall@mail.ccmu.edu.cn)

**Point-by-point responses to the comments from the Editors and reviewers, and a list of changes are provided below.**

(The comments from the Editor and reviewers are listed in blue, which are followed by our responses.)

**Editors**

The authors have described the disease burden of congenital TORCH infections and described their clinical features from a nationwide database. There are considerable methodological constraints which require clarity. The authors need to clarify how the diagnosis of a congenital TORCH infection was made. As has been pointed out by the reviewers as well, the basis of diagnosis of TORCH infection especially that of congenital CMV infection need to be described. Congenital CMV is the commonest congenital infection described in the study and over 50% of the children with congenital CMV infection are beyond the neonatal period. A serological or PCR based diagnosis made beyond 3 weeks cannot differentiate congenital from acquired CMV infection. There are other issues that have been raised by the reviewers and the manuscript requires considerable revision before it can be accepted.

A1: We would like to thank the Editors for all these useful comments. The diagnosis of each of these infections was determined by the corresponding diagnostic criteria established in China. Currently, congenital TORCH infections are usually confirmed by neonatal and maternal serological IgM and IgG antibody testing, and the confirmation of congenital HCMV and HSV infection also requires PCR testing within three weeks in neonates. We have added this description to the text. This manuscript has been substantially revised to address this reviewers’ concerns. The language of the manuscript was also polished by an editing service to improve the readability of the manuscript. Corrections are marked in the revised manuscript.

**Reviewer #1:**

Methods

The authors describe the epidemiology and the disease burden of congenital TORCH infections (Toxoplasmosis, CMV, Rubella, HSV) among hospitalized infants in China. The data has been derived from the FUTURE database which collects data from 27 centres from various regions of China. The authors have stated that the data was on all infants admitted to these hospitals between 2015 and 2020.

Since they have limited their objectives to number of admissions with the TORCH infections, length of stay and treatment cost, this seems reasonable.

However, to make the subsequent data more meaningful, some more background data needs to be given:

1. How were the diagnosis of each of these infections established? Only serological tests or were molecular and/or cultures used?

A1: We would like to thank this respected reviewer for the useful comments. The diagnosis of each of these infections was determined using the corresponding diagnostic criteria established in China.

The diagnostic criteria for congenital toxoplasmosis are stated in the “Clinical use of tests for *Toxoplasma gondii*” officially issued by the National Health Commission of China (formerly the Ministry of Health) (in Chinese) (File download address: http://www.nhc.gov.cn/wjw/s9492/201409/dfb9e7068025408c820e227342f7ac8b.shtml); the diagnostic criteria for congenital toxoplasmosis are stated in the “Diagnosis criteria for rubella” officially issued by the National Health Commission of China (File download address: http://www.nhc.gov.cn/wjw/s9491/200908/42159.shtml); the diagnostic criteria for congenital CMV infection and congenital HSV infection are stated in *Practical Neonatology* and *Expert consensus on standardized TORCH laboratory detection and clinical application* (File download address: http://rs.yiigle.com/CN114452202005/1199015.htm).

Congenital TORCH infections are usually confirmed by neonatal and maternal serological IgM and IgG antibody testing, and the confirmation of congenital CMV and HSV infection also requires PCR testing within three weeks in neonates. We have added this description to the text.

2. Were all the diagnoses made in the newborn period? Especially for CMV, if diagnosis was made after 3 weeks, it is likely to be a postnatal infection. This distinction, to a lesser extent, has to be made for rubella and HSV also. So, the authors have to be categorical that the diagnosis of a CONGENITAL infection was established even though the child may have been admitted at a later age.

A2: Thank you for the kind reminders. As mentioned above, the diagnosis of each of these congenital infections was determined in the newborn period.

3. It is very unclear from the write up if children below 1 year were included or all children ≤ 18 years were included in the analysis. The inclusion criteria says ≤18 years, but the tables all use 29 days- 1 year. The year by year analysis and the month-wise admissions would have little meaning if ALL children ≤18 years were included.

A3: We apologize for the insufficient description. The National Bureau of Statistics of China and the National Health and Health Commission of China define "children" as people under 18 y based on ethnic and demographic characteristics and further stratify them into the following groups: 0–28 d (neonates), 29 d–< 1 y (infants), 1–3 y (toddlers), 4–6 y (preschool children), and 7–18 y (school-age children). The FUTURE database contains data on all children aged ≤18 years. We analyzed hospitalization information for children younger than 1 year of age with congenital TORCH infections; therefore, the descriptions in the table used the groups of 0–28 d and 29 d–<1 y.

4. The exclusion criteria excludes children where data regarding age, gender, burden of disease, diagnosis were not complete. While it makes perfect sense to exclude those with incomplete diagnosis, it would be interesting to see the numbers of those excluded on the basis of lack of other criteria especially if the numbers are large.

A4: We would like to express our appreciation to the reviewer for this thoughtful comment. The exclusion criteria we listed were used in the collection and import steps with the FUTURE database. The excluded FSMRs are limited because the basic registration information requirements have been issued to each member children's hospital during the initial setup. Therefore, we believe that these exclusions have an extremely limited effect on the conclusions of this study.

5. Since the authors have chosen to determine the ethnicity of the patients with infections, it is worthwhile giving the ethnic diversity (Han vs Non-Han) of China as a whole or within the population analyzed. If this is almost the same as the proportion among the infected children, then there is no ethnic variation.

A5: According to the composition of ethnic groups in the seventh population census of the National Bureau of Statistics of China (http://www.stats.gov.cn/tjsj/pcsj/), the ratio of Han to non-Han was 91.11% *vs*. 8.89%, As in our study, the Han to non-Han ratio of hospitalizations was 80.00% *vs*. 20.00% for congenital toxoplasmosis, 92.59% *vs*. 7.41% for CRS, 88.64% *vs*. 11.36% for congenital CMV infection, and 88.89% *vs*. 11.11% for congenital HSV infection, respectively. After performing the statistical analysis, indeed no ethnic variation was observed among the infected children.

Results

To make better sense of the vast data collected, I would like more data given.

1. How many hospitalized children were there in each age group over the 5 years? If we have the number of births/newborns in the 27 hospitals and the number of neonatal diagnosis of the TORCH infections, this would give a better indicator of the burden of the infections.

A1: We agree with your pertinent suggestion. The total hospitalized population was 569,680 in the 0–28 d group and 585,244 in the 29 d–<1 y group. We analyzed the ratios of congenital TORCH infection-related hospitalizations to total hospitalizations within different age groups of patients in the corresponding groups in this manuscript, and the results showed that the ratio of hospitalizations due to congenital TORCH infections was 17.56/100,000 in the 0–28 d group and 12.47/100,000 in the 29 d–<1 y group. As we described in the limitations section of the manuscript, only the FSMRs of hospitalized children were collected in the FUTURE database. However, more detailed data, including the number of births/newborns, were not disclosed in the database, although this issue is interesting and should be analyzed. We also believe that obtaining these data from a larger number of patients will help us to obtain a more comprehensive understanding of the disease burden of TORCH infections.

2. There is a wide disparity in the distribution of hospitals between the regions- 8 in East China and 2 each in South and South-West China. Hence it does not make sense to give the percentage of each infection as a proportion of the total infections in each region. If the population studied in each region is taken as the denominator, then we would be able to glean if an infection is in fact more prevalent in a particular region. Hence, the way this is given in the text and the tables now should be removed or modified.

A2: We appreciate the reviewer for providing useful comments and professional suggestions. We reanalyzed the proportion of patients with each infection to the population studied in each region, and we modified the corresponding data in the text and the tables.

3. As previously mentioned, the season of admission may be valid if only neonatal infections are included. Otherwise, giving this data for all children between 0-12 months gives no meaningful information. A baby born in one particular month could be admitted with cardiac or liver cell failure any time over the next year! If this data is to be included, it should changed to include only newborn. That would give some idea of the seasonality of the infection in the mother.

A3: We agree with the reviewer. We reanalyzed the data in this section, and only neonatal infections were included*.*

Tables and figures:

1. The tables are exhaustive and can be made more brief to give only the relevant information. For example, the clinical manifestations in various organs in CRS (Table 3) in each region is redundant. As previously mentioned, ethnicity would have relevance if the background ethnic make-up is known.

A1: We appreciate the reviewer for providing useful comments and professional suggestions. We modified the tables and further improved the presentation of the data in tables according to your suggestion.

2. The supplementary figures S1 and S2 are too unclear. No details could be made out.

A2: We apologize for the unclear description of Figures S1 and S2. We have modified the data in the figures for a better presentation.

Conclusions

If necessary changes as suggested could be made, the conclusions would be more clear. The authors have clearly identified the limitations of the analysis and have described them. I would like some more detailing on how they expect this data to have public relevance in China.

A: We thank the reviewer for carefully reading our manuscript. This manuscript has been substantially revised in response to the suggestions. We made substantial improvements to the Discussion and Conclusion.

Editorial and Data Presentation Modifications?

More data needs to be presented before the conclusions can be arrived in this study. The language used needs a fair bit of editing before publication.

A: Thank you for this suggestion. We have increased the presentation of the corresponding data in the text and tables, and this manuscript has been substantially revised according to your suggestion. In addition, the manuscript has been polished by native English speakers to improve the description, and the certificate is provided below.

Summary and General Comments

The authors have presented data on congenital TORCH infections among hospitalized children in China over a nearly 6 year period from China. It should give a good idea about the burden of the problem (health and economic) and the prevalence of these infections in China to direct public health measures. I think this would be good data.

However, more background data needs to be given and the analysis needs to be modified to make the results and conclusions more valid.

A: We thank the reviewer for these positive comments. We have thoroughly and carefully revised the manuscript and added the corresponding background data and appropriate analysis to the text and tables according to your suggestion.

**Reviewer #2:**

Methods

This study is a descriptive study, but the field is poorly studied from an epidemiologic standpoint. The true burden of TORCH infections is under characterized and this study adds value into this field. The statistical methods are relatively simple as a descriptive study and appropriate. The data seem to be abstracted from a discharge face sheet which is not clear to me what this contains- I suspect it is akin to a US medical system discharge summary, but the data abstraction should be better described.

A: We would like to thank this respected reviewer for the useful comments. The FUTURE database on which this study is based uses the face sheets of discharge medical records (FSMRs) from subordinate children's hospitals/medical institutions. Basic demographic information, epidemiological characteristics, and descriptions of the clinical diagnosis are generally included in FSMRs. We performed a maximal analysis using the FSMR data and further improved the presentation of the data in tables and graphs. However, a limitation of this study is that the FUTURE database did not include more epidemiological impact indicators.

Tables and figures:

The analysis and the analysis plan matches.

There are some significant limitations in the data- Parvovirus should be excluded as parvovirus has implications in vertical transmission antenatally and can cause hydrops, stillbirth and severe anemia. Post natal admission for parvovirus would be atypical and the authors should exclude it.

A: Hospitalized patients with parvovirus infection were excluded from our included subjects.

It is difficult to believe that there was no vertical transmission of HSV. This is a relatively common TORCH pathogen and certainly exists at higher rates than congential toxo.

A: We thank the reviewer for this suggestion. The epidemiology and disease burden of congenital HSV infection among hospitalized children are described in detail in our manuscript. According to our data, the proportion of hospitalizations for congenital HSV infection cases was 1.05/100,000 patients, and the rate of hospitalization for congenital toxoplasmosis was 0.70/100,000 patients.

Conclusions

In the conclusions, the authors perform a comparative analysis of different TORCH infections. This is likely inappropriate due to the wide variety in clinical sequelae, clinical presentation and antenatal route of infection.

A: We thank the reviewer for carefully reading our manuscript. We have thoroughly and carefully revised the Discussion and Conclusion according to your suggestion.

In the section on CRS, additional information on maternal immunization history and rubella susceptibility in the setting of the described outbreak is warranted. Vaccination is a well described strategy to eliminate CRS and should be commented on.

A: We appreciate the reviewer for providing useful comments and professional suggestions. We have included this vital information in the CRS section, and this section has been substantially revised in response to these comments.

The authors should describe the epidemiology of postnatal serology and the difference in serologic titers (particularly positive IgG) and congenital TORCH infection diagnosis. Both background on transplacental passage and stability of IgG should be described as well as a description on the utility of serology for postnatal screening as this is elucidated in several areas.

A: We would like to thank this respected reviewer for the useful comments. As we described in the limitations section of the manuscript, only the FSMRs of hospitalized children were collected in the FUTURE database. We acknowledge that the description of seroepidemiology is an important and highly regarded issue, and we also believe that obtaining these data from a larger number of patients will help us obtain a more comprehensive understanding of the utility of serology for postnatal screening. In fact, we are continuing to expand the content of the FUTURE database. We believe that in the next five-year update, the statistics will incorporate more epidemiological impact indicators such that the resulting data will be more convincing and authoritative.

Several limitations of this study should be discussed in the conclusions including the inability to detect mildly symptomatic infections for all TORCH pathogens as well as the loss of infants who died secondary to their infection or fetal stillbirth. While this dataset is extremely important and adds to the data, it underrepresents the epidemiologic burden of TORCH and this should be explicitly stated.

A: We appreciate the reviewer for carefully reading our manuscript and providing useful comments and professional suggestions. We substantially improved the description of the limitations in response to the suggestion.

Editorial and Data Presentation Modifications?

The introduction is very short and largely written in a matter of fact matter without a lead into the subject material and study desigen. I would recommend additional background and information should be included to introduce and link the study.

A: We apologize for the incomplete description in the Introduction. We have added the details regarding this issue and made substantial improvements to the Introduction according to the suggestion.

Summary and General Comments

The manuscript by Dr. Zhang and colleagues about the epidemiology and burden of TORCH infections is a novel study that substantially adds to information in the field on this topic. The study design is a retrospective collection of data from children age 0-1 with a diagnosis code of a congenital infection. They identified 173 patients from a wide and diverse geographic region in China from their database. They then go to describe the distribution attributable to different pathogens and some information on the sequelae of infection.

A: We appreciate the reviewer for carefully reading our manuscript and providing useful comments.

This manuscript would benefit from additional detail regarding serologic screening, background on the diagnosis of vertical transmission versus postnatal transmission (well described in CMV for example) as well as an additional figure with the geographic region of each infection (as this is an epidemiologic study and local geopolitical and cultural risks may contribute to disease prevalence).

A: The diagnostic quality control measure for including patients in the FUTURE database is very stringent; all the patients met the corresponding diagnostic criteria promulgated by the Health Commission of the People’s Republic of China (formerly the Ministry of Health) and *Expert consensus on standardized TORCH laboratory detection and clinical application*. As you suggested, this issue is vital that we are investigating with all of our member children's hospitals. We believe that in the next five-year update, the statistics will incorporate more epidemiological impact indicators.

As suggested, we supplemented the analysis of the geographic distribution of each infection, and we improved the presentation of the data in the tables and graphs.
